# South African users’ function and experience with a magnetorheological microprocessor knee: A mixed methods study

**DOI:** 10.33137/cpoj.v8i1.45286

**Published:** 2025-06-12

**Authors:** S Visagie, B Theron

**Affiliations:** 1 University of Stellenbosch, Division of Disability and Rehabilitation Studies, Faculty of Medicine and Health Sciences, South Africa.; 2 Össur South Africa, Cape Town, South Africa.

**Keywords:** Amputation, South Africa, Lower Middle-Income Countries, Prosthetic Prescription, Rheo XC, Transfemoral, Transtibial, Rehabilitation, L-Test, Microprocessor Knee, Knee Disarticulation, MPKs

## Abstract

**BACKGROUND:**

Microprocessor knees (MPKs) support safe and confident prosthetic walking. Their cost often prohibits prescription in low- and middle-income settings like South Africa. Funding of high-end prosthetic products in South Africa is dependent on justifications that explain why the component is prescribed, and how it can improve the user`s function. There is little local evidence to support these justifications.

**OBJECTIVE:**

To explore and describe South African users’ function and experience with the Rheo XC microprocessor knee (MPK).

**METHODOLOGY:**

An explanatory sequential mixed methods design was used. A pre-test, post-test study was followed by a descriptive qualitative study to explore and explain the observed outcomes. In the pre-test phase, baseline data were collected while participants used their regular non-microprocessor knees (e.g., mechanical or hydraulic joints). Post-test data were collected after a two-week trial with the Rheo XC knee joint. Data were collected from 16 consecutively sampled participants, using a self-developed functional level scale and the L-Test. Nine (56.3%) participants had a transfemoral amputation, six (37.5%) had a knee disarticulation and one (6.3%) had bilateral amputations (transtibial and transfemoral). Baseline and follow-up data were paired for each participant and analyzed with the Wilcoxon Signed-Rank test. The descriptive qualitative study explored six purposively sampled participants’ experiences of the trial knee through semi-structured interviews. Inductive thematic analysis was done.

**FINDINGS:**

The time to complete the L-Test decreased on average 7.5 s between baseline (35.4 s) and post-test (27.9 s) data. L-Test Wilcoxon Singed-rank findings showed a significant increase in walking speed (p < 0.001). Mean functional level scores increased by an average of 12.7 points (p < 0.001) with improvements observed across all activities except running, for which scores remained unchanged. Two themes emerged from the qualitative data. Theme 1: *Acceptance of the MPK* showed enthusiasm for the MPK. However, Theme 2: *Real-world limitations of the MPK* cautioned that the MPK is not suitable for everybody.

**CONCLUSION:**

This study provides context specific scientific evidence that may support funding decisions for MPKs in South Africa. However, it is not suitable for everyone, and a trial period to assess appropriateness is advised before prescription. The test period in this study was short, and further research over longer durations is recommended.

## INTRODUCTION

Persons with amputations expressed the notion that prostheses “normalize” their functionality and physical appearance.^1,2^ Over the years, prosthetic development has aimed to enhance this sense of “normalization” and minimize functional loss, benefiting from advanced materials and the integration of software technologies. An example is the use of sensors and microprocessor-controlled software in prosthetic knee and ankle components.^3^

Microprocessor knees (MPKs) provide high levels of safety, consistency, and confidence during walking.^3^ One of the most widely recognized benefits of MPKs is their ability to reduce stumbles and falls.^4^ Research has also shown improved physiological functions such as a decrease in oxygen cost when walking with MPKs compared to non-microprocessor knees (NMPKs).^5^ MPKs enhance walking speed, stair climbing, hill descent, walking on uneven terrain, and the ability to multitask while walking.^5^ However, MPKs are expensive, with prices starting at over R600,000 (US$32,000) and reaching up to R1 million (US$55,000).

Even though research shows that future savings and financial benefits might offset the initial high cost of the MPK^6,7^ the expense remains a barrier to prescription.

Provision of prosthetic components—whether costly or not—is hampered by a shortage of financial resources, poor social security systems, and little health insurance in low-and middle-income countries (LMICs).^8,9^ Service-related factors such as limited access to equipment and materials, limited numbers of adequately trained prosthetists, clustering of prosthetic services in metropoles and bureaucratic red tape further obstructs prosthetic services.^8–13^ There is also limited awareness of services, service pathways, and device options among users.^8,9,14^ Geographical challenges, large rural areas, inadequate transport systems, and poor infrastructure further decrease access to prosthetic devices.^8,10,14–16^ Finally, prosthetic components dependent on computer software such as MPKs are sometimes considered too fragile for the harsh environments and employment requirements in LMICs.^10^ Therefore, context specific research is required to ensure that products which has shown superior function in Global North settings are appropriate for use in the LMICs.^10,14^

The only statistics currently available on the need for prosthetics in South Africa comes from the 2022 national census which states that there are around 430,000 upper and lower limb prosthetic users in the country.^17^ Waiting periods^9,15,16,18^ and waitlists for prostheses^19^ are long. In addition, while a prosthesis is often promised at the time of amputation^20^ receiving one is not a given.^21,22^ For those who do receive a prosthesis, functional ability is often not optimally supported by the componentry provided in both the private^9^ and public sectors.^15^

In the South African private sector, the funding of high-end prosthetic products, including MPKs, is dependent on special justifications explaining the functional benefits of the more expensive component, and lengthy waiting periods before authorization for the components are received.^9^ MPKs are seldom covered by medical insurance, but are provided through the Road Accident Fund.^9^ In the public sector, which provides the bulk of prosthetic services in the country and will play an even larger role after the implementation of the National Health Insurance Act, high-end products are not currently funded.^15^ Prosthetic component selection is based on empirical knowledge and financial considerations rather than evidence-based guidelines in both the government and private sectors. Inconsistencies, over, and under prescription, which are at least in part due to insufficient local evidence, are real concerns.^9^

Therefore, the aim of the study was to explore and describe users’ function and experience with an MPK, the Rheo XC, which generates knee joint resistance through a magnetorheological clutch mechanism^23^ in comparison to the NMPKs participants normally use in South Africa. It is hoped that the results will contribute to the evidence base for prosthetic practice in South Africa. The study focused on a specific microprocessor knee (MPK) because, although MPKs are similar in type, they differ in the specific functional features they provide.^24^

## METHODOLOGY

To ensure robustness and rigor, an explanatory sequential mixed methods design was employed. Changes in user function were determined using a quantitative pre-test, post-test design. Functional changes were contextualized and further explored with a qualitative descriptive study. The results were analyzed separately and triangulated in the discussion.^25^

### Pre-test, post-test

Sixteen consecutively sampled prosthetic users were included in the pre-test, post-test study. They had to:

- Be older than 18 (age of majority in South Africa)
- Have a transfemoral amputation or knee disarticulation
- Have an amputee activity level of K2-K4 on the AMPPRO.^26^
- Use a prosthesis for more than four months to ensure sufficient adaptation to the prosthesis.^27^

Users who did not complete the two-week trial period or who experienced a health crisis such as a stroke that might influence their functioning, during the two weeks, would have been excluded. However, these situations did not occur. Four users who started the trial did not complete the study (one had a faulty MPK and the other three did not complete the post-test). Baseline and follow up data on functioning and walking speed with the participant`s conventional knee and the Rheo XC were collected between 01 March 2023 to 31 January 2025. The pre-test was completed before the Rheo XC was fitted, and post-test data were collected two weeks after fitting the device.

History, maturation, cognitive learning, interrater reliability, statistical regression, and sensitization all pose threats to the internal and external validity of the pre-test, post-test design.^28^ These challenges of one group designs are acknowledged. However, in this study, with its short time frame and contained independent variable (i.e. a different knee component), many of these concerns were mitigated. External changes should not influence the function with the knee because the physical environment in which the user operated remained the same. To prevent maturation effects, users had to have been walking with a prosthesis for four months or more at the time of the pre-test. Function is physical in nature; thus, cognitive learning was not a concern. Spontaneous remission does not pose a risk to validity as an amputation is a permanent impairment. Factors such as interest level, and general fatigue could not be controlled and might have influenced scores. Clinical evaluation commonly involves pre-test, post-test assessments.^29^ Thus, this design was deemed suitable for the current study with its focus on clinical application. In addition, results were verified through triangulation with qualitative descriptive findings.

The supplier of the Rheo XC, Össur, offers trial periods with the knee to users in South Africa (this service has been offered for years; it was not started with the current study in mind). The current study utilized data collected during this trial period. Prosthetists request trial units for users based on the user's function and physical ability. During the study period, the prosthetist was informed about the research only after a trial request for the MPK knee being studied was received. They were asked to provide users with the information leaflet and informed consent form, and to obtain written consent from those willing to participate in the study. Participation was voluntary, and the informed consent form emphasized that the decision to participate was entirely up to the user. The decision did not affect access to a trial MPK, or any other service provided to them by the company or prosthetist. All users who trialed the MPK during the study period were approached consecutively to participate in the study. Twenty agreed to participate, of whom 16 completed the study. The number of users who requested a trial period and who were approached to participate is unfortunately not known. This omission and the small sample size reduced internal validity and generalizability of the findings.

Data were collected using a functional level scale and the L-Test of Functional Mobility (L-Test). The functional level scale was developed though combining The Trinity Amputation and Prosthesis Experience Scales – Revised (TAPES-R),^30^ the Locomotor Capabilities Index-5 (LMCI5)^31^ and the Prosthetic Limb Users Survey of Mobility (PLUS-M).^32^ These scales assess slightly different functional aspects and through combining them a comprehensive picture of changes in functional ability could be obtained. All three of these scales are valid and reliable.^30–32^ However, the combination used in this study was not tested for reliability and validity. This is a limitation that can negatively affect the study's reliability and external validity. Questions focused on indoor and outdoor mobility, as well as participation in community, sport, and work activities. Scoring was based on a five-point Likert scale, with total scores ranging from 20 to 80. Higher scores indicated better function.

The L-Test is a simple and quick mobility test. It measures the time to get up from a chair (seat height 46 cm), walk 3 meters, turn 90°, walk 7 meters, turn 180°, and walk back to the chair in seconds.^33^ The L-Test has shown high levels of inter- and intra-rater reliability (0.97 and 0.97 respectively) as well as concurrent validity.^33^ It also has a low ceiling effect in higher functioning prostheses users.^33^ Prosthetists measured the L-Test times with a smart phone stopwatch. In addition, data on demographic variables, amputation details, and the prosthetic components habitually used were collected and coded. Items that were scored as “not applicable” were removed from the pre- and post-test scores for that specific participant. Descriptive analysis was done after which the Wilcoxon Signed-Rank test was used to evaluate for significant differences in function and L-Test scores between matched pairs.

### Qualitative descriptive study

During an iterative process, six prosthetic users who had completed the trial period were purposively sampled using a maximum variation strategy.^34^ Variables used to ensure maximum variation included gender, age, level of amputation, K-Level, differences in L-Test and functional level scores. Data were collected with a 30-minute audio recorded, telephonic, semi-structured interview. Interviews were guided by an interview schedule developed by the authors. Questions focused on the participants’ opinion of the Rheo XC and their thoughts on why their function and L-test scores were different or similar in the pre- and post-test. Inductive thematic analysis was done.^35^

The first author identified codes (meaningful parts, ideas, and key concepts in the data), through line-by-line coding, and provisional themes manually, as interviews were completed. Provisional themes and questions raised by the analysis informed further sampling, data collection and analysis. Data saturation was reached after the 6^th^ interview. Codes and themes were finalized and verified by the second author. Trustworthiness is supported by purposive sampling, using an interview schedule, data saturation, a second person verifying themes and describing participants’ demographic details. An additional limitation of the study is the absence of independent coding and consensus-building between the two authors, which would have enhanced trustworthiness.

### Publication ethics

The study was approved by Stellenbosch University's Health Research Ethics Committee (N22/08/097). The risk for physical harm during the test period was very low since the participants had been walking with the prosthesis for at least four months and had a minimum ability to walk in the community on level surfaces as per inclusion criteria.

## RESULTS

### Quantitative

Of the 20 participants who agreed to take part, 16 completed the study, of whom 13 (81.25%) were men. Nine (56.25%) had transfemoral amputations and functioned at a K3 level (**Table 1**). For four participants with knee disarticulation, a low-profile foot was required to accommodate the length of the Rheo XC knee. The reasons for the foot changes in the other two cases were unclear. These changes negatively affect the internal validity of the study.

**Table 1.** Demographic and amputation related information of participants (n=16).

		No. (%)	
**Gender**	Men	13 (81.3)	
	Women	3 (18.8)	
**Cause of Amputation**	Vascular	3 (18.8)	
	Trauma	11 (68.8)	
	Cancer	1 (6.3)	
	Congenital	1 (6.3)	
**Level of Amputation**	Transfemoral	9 (56.3)	
	Knee Disarticulation	6 (37.5)	
	Bilateral (TF & TT)	1 (6.3)	
**K-Level**	K2	2 (12.5)	
	K3	9 (56.3)	
	K4	5 (31.3)	
	**Pre-Test**	**No. (%)**	**Post-Test**
**Prosthetic Knees**	Paso Knee (Ossur)	4 (25.0)	Rheo XC
	3R31 (Otto Bock)	1 (6.3)	
	3R106 (Otto Bock)	2 (12.5)	
	3R80 (Otto Bock)	3 (18.8)	
	3R95 (Otto Bock)	1 (6.3)	
	3R78 (Otto Bock)	1 (6.3)	
	OHP5 (Ossur)	1 (6.3)	
	Aspire H1 (Ossur)	1 (6.3)	
	Balance Knee (Ossur)	1 (6.3)	
	Total 2100 (Ossur)	1 (6.3)	
**Prosthetic Feet**	Vari-Flex (Ossur)	5 (31.3)	Unchanged
	Triton (Otto Bock)	1 (6.3)	Changed to Pro-Flex LP
	Balance J (Ossur)	1 (6.3)	Changed to Breeze (Steeper)
	Trias (Otto Bock)	2 (12.5)	Changed to Pro-Flex LP
	Taleo (Otto Bock)	1 (6.3)	Unchanged
	1D10 (Otto Bock)	1 (6.3)	Unchanged
	SACH (Otto Bock)	1 (6.3)	Changed to Pro-Flex ST
	DP Flexion (Ossur)	1 (6.3)	Changed to Pro-Flex ST
	Pro-Flex ST (Ossur)	1 (6.3)	Unchanged
	Triton Sideflex (Otto Bock)	1 (6.3)	Unchanged
	Breeze (College Park)	1 (6.3)	Unchanged

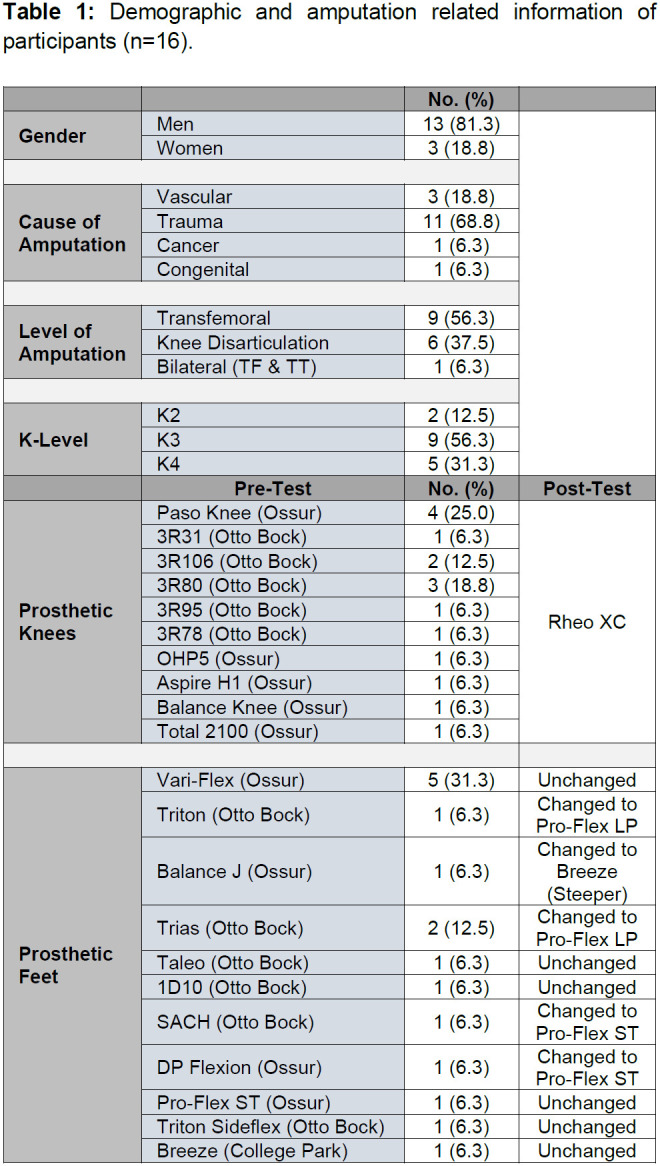

Their average age was 51.9 (SD = 14.9) ranging from 26 to 74 (**Table 2**). Participants completed the L-Test on average 7.5 s faster in the post-test than in the pre-test. The mean function score was 12.7 higher in the post-test than the pre-test (**Table 2**).

**Table 2.** Descriptive summary of age, pre-test, post-test, L-Test and function scores.

	Low	High	Mean (SD)	Cohen's d	Z-value	P-value
**Age**	26	74	51.9 (14.9)			
**Pre-Test L-Test (s)**	17.5	71.0	35.4 (16.4)	0.52	−3.52	0.00
**Post-Test L-Test (s)**	14.0	62.2	27.9 (12.3)			
Pre-Test Function^*^	31	76	52.7 (12.2)	1.22	−3.41	0.00
Post-Test Function^*^	49	78	65.4 (8.3)			

* Higher scores indicate better function.

**Figure 1:**
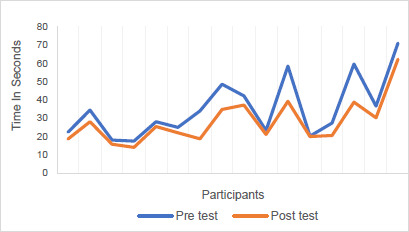
L-Test times before and after the intervention.

L-Test scores were consistently lower in the post-test indicating faster walking and turning speeds with the Rheo XC (**Figure 1**). This difference varied from 0.3 s to 20.9 s. The Wilcoxon signed-rank test of matched L-Test data showed a Z-value of −3.52 and p < 0.001.

**Figure 2:**
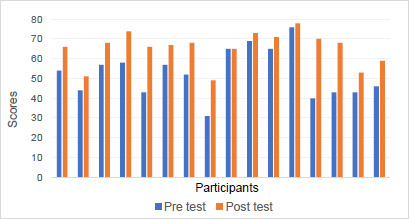
Functional scores before and after the intervention.

**Figure 2** shows improved function with the Rheo XC. The Wilcoxon-Signed-Rank Test found a z value of −3.41 with p < 0.001.

**Figure 3:**
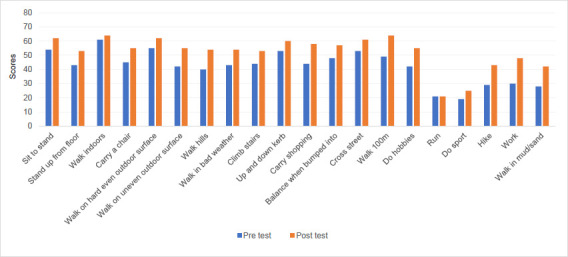
Comparison of individual functional scores. Activities with no applicable scores included run (1), hobbies (1), walk in bad weather (2), sport (2), work (2), and walk in mud/sand (3). Numbers in parentheses indicate the number of participants who marked the activity as “not applicable.”

Higher scores indicate better function. All but one activity (running, which remained the same) scored higher in the post-test than the pre-test (**Figure 3**). The variables of work (18 points), walk 100 m (15 points), carry shopping, walking hills, walking in mud/dust (14 points), hiking, and walking on uneven outdoor surfaces (13 points) showed the biggest positive change. All participants indicated that it would be easy to charge the battery.

### Qualitative

Among the six participants one was a woman, and one had an amputation due to vascular reasons. Their age ranged between 26 and 74. L-Test speed differences ranged from 0.2 s to 19.3 s. The differences in functional scores ranged between 2 and 16 (**Table 3**).

**Table 3:** Demographic and prosthetic characteristics of participants in the qualitative phase.

Subject	Gender	Cause of Amputation	Age	K level	Amputation Level	Foot (Pre-test & Post-test)	Pre-test knee	L-Test speed difference (m/s)	Function difference
**P1**	Male	Trauma	35	K3	KD	Triton & Pro-Flex LP	3R31 (Otto Bock)	6.4	7
**P2**	Male	Congenital	31	K3	KD	Triton Sideflex	3R80 (Otto Bock)	0.2	2
**P3**	Male	Trauma	26	K4	Bilateral	Vari-Flex	Paso knee (Ossur)	10.5	10
**P4**	Male	Trauma	65	K4	KD	Trias & Pro-Flex LP	3R80 (Otto Bock)	3.4	16
**P5**	Female	Trauma	74	K4	KD	Balance J &Breeze)	3R106 (Otto Bock)	15.7	11
**P6**	Male	Vascular	68	K2	TF	Pro-Flex ST	Aspire H1 (Ossur)	19.3	6

Participants were enthusiastic about the Rheo XC, as illustrated by Theme 1: Acceptance of the MPK. However, concerns were raised as well, indicating that MPK is not suitable for everybody, as explained in Theme 2: Real-world limitations of the MPK.

### Theme 1: Acceptance of the MPK

Participants found the Rheo XC safe, easy to walk with, and responsive to their mobility needs. *“It was immediately comfortable. I felt safe. The knee became my own, in other words, like my living knee. That was what it felt like. The quick responses…I liked it. It is an absolute pleasure to put it on. The control of the knee gives you many options. You can walk faster with confidence. The knee gives that to you, the confidence….I did not want to give it back!”* P6

Participant 1 felt that the Rheo XC joint allowed a more natural walking pattern which he thought led to an increase in walking speed. *“It mimics natural walking…It is definitely a more natural walking pattern, which result in me being able to walk faster. Even turning on my left leg [prosthetic side] was very natural.”* P1

The quick response of the joint to changes in walking speed and/or direction aided a normal walking pattern. “*I can move faster. I can turn easier. It is easier to pick up stuff, especially large items. On uneven ground, much safer, sturdy. You are so confident. You can walk like you want to. You do not have to worry about stepping on a pebble, or a clump of grass. You walk; this is just fantastic.*” P4

Even though Participant 2's L-Test results showed only a 0.2 second difference (between pre- and post-test data), he was more satisfied with the new joint due to its agility, lower energy consumption, and better support of the residual limb. “*After walking on the microprocessor knee for two weeks it was difficult to go back to the hydraulic knee. I will not call it a shock, but I quickly realized how much I used the functions of the microprocessor knee. My brain adapted quickly to everything the microprocessor offered me in comparison with my other knee…The swing through of the knee was just easier. Overall, I required less energy to walk with the knee. The knee helped a lot to decrease the effect and strain on my stump. I felt less tired at the end of the day.*” P2

Participants extolled how safe they felt using the Rheo XC joint. “*It is very safe. You feel safe on it. It will not give way under you. You have confidence. You do not have to be careful to prevent a fall. That [the guardedness against falling] is gone. It gives you confidence to walk and you can walk faster. It is the best leg I ever had… You have more control, balance and confidence. For sure. When you stand you stand solidly.*” P4

These advantages translated into improved functionality. “*I like fishing, I am next to the water, big clumps of grass, uneven ground. I do not want to place the other knees in a bad light, but if you put your weight wrongly on the foot it gives in. Not this one. If there is weight on it, it is solid. That is a huge benefit.*” P4

Participant 6 felt a waterproof knee will enhance his functionally further. “*I would have liked it to be waterproof. And that I can walk in the sand, in the swimming pool. In the sea… with my grandchildren in the shallow water*.” P6

### Theme 2: Real-world limitations of the MPK

The weight of the Rheo XC in relation to user strength must be considered during prescription. “*The weight, it was extremely heavy. After the first 2 days I thought my left hip was dislocated*.” P5

“*Obviously, the weight of the knee one can feel it immediately. Even though there is less strain on your [remaining] knee you can tell you are walking with a prosthesis or a knee that is much heavier.”* P2

The size of the Rheo XC adds to the overall length of the prosthesis, which can be problematic for shorter people or those with long residual limbs. “*I am short, so the knee with its fixed length and the foot that was at its lowest was still longer than my real right leg*.” P5

Participants felt the battery life was short. “*I walked through the shops through the day then it starts peeping at me. I cannot understand why it is peeping. Then I see the battery is going flat.*” P4

Another challenge that was raised was that the knee can hamper driving a vehicle as it might interfere with the pedals. “*You struggle in a car because it just wants to go forward. Then it is in the way of the pedal, the accelerator…you just lift your foot slightly and ‘zoep’ [local slang for something happening without warning] it wants to straighten*.” P4

Thus, for a successful prescription, knee characteristics must match user abilities. To ensure challenges are identified and the MPK is prescribed appropriately, a trial period is recommended. “*The fact that there is a trial period is very good. Walk with the knee. See what it does for you. Is it sufficient for your needs? And if you feel it works for you, you have compared it with other knees and feel it ticks more boxes for you in your daily activities I will recommend it rather than a hydraulic or polycentric knee.*” P2

## DISCUSSION

This mixed methods study explored and described South African users’ function and experience with the Rheo XC knee. Both user function and L-Test scores showed improvement from pre-test to post-test. The positive impact of the knee joint was further supported by qualitative data.

L-Test scores exceeded both the normative values and the minimum detectable change (MDC95), which is the smallest improvement needed to be 95% confident that the change is clinically meaningful. The L-Test norm is 41.7 s ± 16.8.^33^ The pre-test score was 6.3 s below this norm and the post-test score was 13.8 s below the norm. This might be due to the normative data being from 2005. The advancement of prosthetic components over the last 20 years should impact walking speed positively. The L-Test MDC95 for persons with transfemoral amputations are 2.9 s.^36^ Current data showed that 11 (68.75%) participants’ scores decreased with more than 2.9 s, with an average decrease of 7.51s across the group. From this it can be concluded that the change in scores is clinically meaningful and infers a change in ability rather than measurement error.^37^

The reduction in L-Test time in the current study was similar to what was documented in previous similar studies. Davie-Smith and Carse (2021)^38^ found that L-Test scores decreased on average with 5.15 s after 6 months. Participants in the study by Davie-Smith and Carse (38) used a variety of MPKs, with the most common being the Kenevo and various versions of the C-Leg. Howard et al (2018)^29^ found a mean decrease of 7.4 s among three participants after switching from a NMPK to the Rheo Knee 3. They indicated that the L-Test score for the other four participants was inconclusive without providing a mean for L-Test score change across the group.

Functional level scores also improved significantly. Previous research on the Ottobock C-Leg^®^ and the 3E80, which features a microprocessor-controlled stance-swing phase switch, has similarly shown improvements in functional performance^39^ and walking speeds.^40^

The clinical value of the magnetorheological MPK was further supported by qualitative data, with participants reporting a positive experience using the device. In their opinion decreased energy use, a more fluid walking pattern, as well as improved agility, and safety translated to higher walking speeds. Decreased energy consumption,^5^ increased agility^5^ and safety^3,4,41^ has been identified as advantages of MPKs in previous research. For some participants, the Rheo XC came close to replicating the function of their natural knee—an outcome that previous studies have shown users desire from their prostheses.^1,2^ The results showed that participants experienced important clinical benefits compared to their non-MPK knee.

Higher-level functions such as working, walking 100 meters, carrying shopping, and walking on uneven surfaces and hills showed the greatest improvement. This might be due to the safety features of the Rheo XC, which allows movement, weight shift, turning, and change of direction without fear of falling as described by participants during the interview and shown in previous studies.^4,7^ At the same time activities requiring speed and high levels of agility such as running and participating in sport remained a challenge for many.

Although concerns have been raised that African conditions might be too harsh for an MPK, the current short-term findings did not support this. However, longer-term studies are needed to confirm its durability and performance. Most participants indicated the ability to function on uneven surfaces and in mud and dust. Qualitative findings provide examples of walking effectively in pastoral areas and pursuing outdoor activities. All participants had access to electricity to charge the battery. Although a longer battery life would be beneficial.

With these functional gains and previous research showing the long-term economic benefits of MPKs,^6,7^ it is important that the South African government and other funders of prosthetic components in South Africa consider MPKs alongside other prosthetic knees. The initial monetary outlay might be compensated for by savings in the long run and better user function. Savings occur because MPKs have a longer life cycle than mechanical knees as reported by Kuhlman et al,^7^ come with a warrantee (two years in the case of the Rheo XC knee), of which the cost is included in the initial price of the MPK,^7^ that guarantee free of charge maintenance, and reduces falls and thus costs incurred because on injury.^6,7^ The economic benefits of better function and a wider range employment options have not been researched.

While quantitative findings indicate that Rheo XC knee joint improved function for all the participants, qualitative data showed that it was not the most optimal solution for all. The size of the joint makes it unsuitable for a shorter person with a through knee amputation or with a long transfemoral residuum. The weight was also concerning to some as also shown previously.^38^ Lighter MPKs or NMPKs might be more suitable for frail users or users with weak residual leg muscles. These findings support the notion that prosthetic knee prescription should be based on user characteristics and functional needs. Ideally users should be offered trial periods with different components before a knee is prescribed.

The study results must be interpreted against the limitations discussed in the methods section and summarized here. The pre-test, post-test design suffers from a lack of a control group. The reasons for choosing this design, along with potential challenges, are outlined in the Methods section.^28,29^ These challenges were further mitigated by triangulating the pre- and post-test results with qualitative findings.^25^ The small number of participants, lack of information on users whose trial requests were rejected, and the need to change the foot in some instances to a low profile foot reduce the generalizability and internal validity of the results. The combined data collection tools were not tested for reliability and validity. Additionally, interrater bias may have occurred during the L-Test, as different prosthetists timed different users. While the second author verified the codes and themes, independent coding and consensus-building were not conducted during the qualitative data analysis.

## CONCLUSION

This study provides context specific evidence that may support funding of the magnetorheological MPK in South Africa. However, it is not suitable for everyone, and a trial period to assess appropriateness is advised before prescription. This evidence may benefit South African users, providers, and funders by assisting in the selection and prescription of appropriate knee components. Research is recommended to determine whether the improved functioning supported by MPKs translates into enhanced employment opportunities and income generation.
